# Editorial: Advancements in tertiary lymphoid structures: insights for cancer treatment

**DOI:** 10.3389/fimmu.2026.1965697

**Published:** 2026-08-26

**Authors:** Jing Xu, Xiangjie Di, Xiao Liang

**Affiliations:** 1 Department of Obstetrics and Gynecology, Chongqing Health Center for Women and Children, Women and Children’s Hospital of Chongqing Medical University, Chongqing, China; 2 Cancer Center, Clinical Trial Center, National Medical Practice Administration (NMPA) Key Laboratory for Clinical Research and Evaluation of Innovative Drug,West China Hospital, Sichuan University, Chengdu, Sichuan, China; 3 Key Laboratory of Obstetrics and Gynecologic and Pediatric Diseases and Birth Defects of Ministry of Education, West China Second Hospital, Sichuan University, Chengdu, Sichuan, China

**Keywords:** cancer immunotherapy, cancer-associated fibroblast, tertiary lymphoid structures, tumor microenvironment, tyrosine kinase inhibitor

Tertiary lymphoid structures (TLS) are ectopic lymphoid aggregates that form within chronically inflamed or neoplastic tissues, where they support antigen presentation, lymphocyte priming, and adaptive anti-tumor immunity. Mounting evidence links mature, intratumoral TLS with favorable prognosis and improved response to immune checkpoint inhibitors. Long considered a pathological curiosity, TLSs now serve as a promising biomarker and therapeutic target. Researchers still face major challenges in understanding TLS seeding, establishing robust measurement approaches, and translating TLS-based strategies into therapy.

In this Research Topic, we gathered five contributions that map the field from cellular origin to clinical translation. Among the contributions, Barnes et al. combined a murine melanoma model in which TLS form spontaneously with Cxcl13 reporter mice and single-cell RNA sequencing to delineate seven cancer-associated fibroblast (CAF) subsets, identifying a CXCL13+ population as the “central organizer” that seeds B-cell aggregation. Crucially, ablating these CXCL13+ CAFs abrogated B-cell accumulation and accelerated tumor growth—providing, to our knowledge, the clearest causal evidence that fibroblasts, rather than hematopoietic cells, issue the chemokine cue that builds TLS. Two studies then convert this biology into clinically actionable metrics. Zhang et al. combined TLS density and tumor budding in a cohort of 107 patients with synchronous colorectal cancer liver metastases, together with an external validation set. The combined index achieved an AUC of 0.89 for survival prediction, superior to each single marker. While this represents a practical histopathological tool, it was derived from synchronous-metastasis patients and needs validation in broader cohorts.

Complementing this tissue-based approach, Li et al. build a non-invasive MRI-clinicoradiologic model that preoperatively identifies “MT-HCC” (microvascular invasion-positive, TLS-positive hepatocellular carcinoma). In exploratory hypothesis-generating analyses, patients receiving postoperative tyrosine kinase inhibitor-immune checkpoint inhibitor (TKI-ICI) therapy exhibited favorable recurrence-free survival (RFS) outcomes. Accordingly, Li et al. proposed that a pre-existing TLS-rich microenvironment may sensitize tumors to immunotherapy, although this observation awaits validation in prospective studies. The breadth of TLS relevance is captured by Bai et al., whose review synthesizes TLS biology in head and neck cancer, compares detection methods from histopathology to spatial transcriptomics, and enumerates therapeutic strategies (LTbetaR agonism, local chemokine delivery, vaccine or CAR-T combination). Finally, Nakamura et al. studied enzootic bovine leukosis. Exhausted CD4^+^ T cells and tumor-derived B cells in this setting overproduce CCL4, which recruits monocytes that develop into immunosuppressive macrophages with impaired antigen presentation. These data uncover a chemokine-macrophage axis that blunts TLS-based immunity and provides cross-species insight into microenvironmental immune restraint.

Taken together, standardized scoring systems for TLS that account for tissue maturity and local-tissue context are still under development, while non-invasive approaches to monitor TLS dynamics during treatment are in their early stages. An important open question remains regarding causal relationships: whether therapeutic induction and maturation of TLS can directly drive clinical benefit, or whether TLS simply serve as valuable biomarkers of pre-existing anti-tumor immune activity. We believe this topic synthesis can offer useful references for future investigators. With joint efforts from authors, reviewers and editors, we hope this Research Topic consolidates current understanding and helps shape future research directions for TLS-oriented cancer immunotherapy.

